# Better-than-chance prediction of cooperative behaviour from first and second impressions

**DOI:** 10.1017/ehs.2023.30

**Published:** 2024-01-08

**Authors:** Eric Schniter, Timothy W. Shields

**Affiliations:** 1 Economic Science Institute, Chapman University, Orange, CA 92866, USA; 2 Center for the Study of Human Nature, California State University Fullerton, Fullerton, CA 92831, USA; 3 Argyros School of Business and Economics, Chapman University, Orange, CA 92866, USA; 4 Division of Anthropology, California State University Fullerton, Fullerton, CA 92831, USA

**Keywords:** cheater detection, cooperation prediction, Prisoner's Dilemma, photographs, thin-slice video

## Abstract

Could cooperation among strangers be facilitated by adaptations that use sparse information to accurately predict cooperative behaviour? We hypothesise that predictions are influenced by beliefs, descriptions, appearance and behavioural history available for first and second impressions. We also hypothesise that predictions improve when more information is available. We conducted a two-part study. First, we recorded thin-slice videos of university students just before their choices in a repeated Prisoner's Dilemma with matched partners. Second, a worldwide sample of raters evaluated each player using videos, photos, only gender labels or neither images nor labels. Raters guessed players’ first-round Prisoner's Dilemma choices and then their second-round choices after reviewing first-round behavioural histories. Our design allows us to investigate incremental effects of gender, appearance and behavioural history gleaned during first and second impressions. Predictions become more accurate and better-than-chance when gender, appearance or behavioural history is added. However, these effects are not incrementally cumulative. Predictions from treatments showing player appearance were no more accurate than those from treatments revealing gender labels and predictions from videos were no more accurate than those from photos. These results demonstrate how people accurately predict cooperation under sparse information conditions, helping explain why conditional cooperation is common among strangers.

## Graphical abstract

**Figure:**
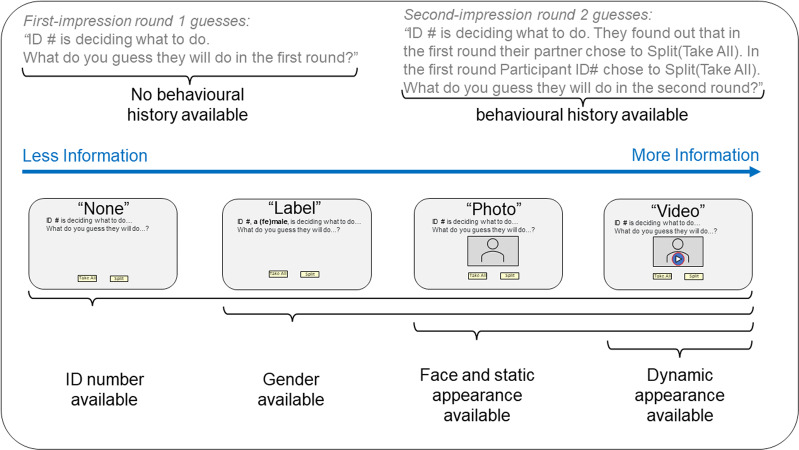

**Social media summary:** People can predict others’ cooperative behaviours in a repeated Prisoner's Dilemma with better-than-chance accuracy.

## 1. Introduction

Opportunities for cooperation with strangers and repeated interaction have presented recurrent adaptive problems throughout human evolutionary history (Fehr & Henrich, 2003). Potentially valuable interactions with strangers entail danger, exploitation and mistrust (Daly & Wilson, 1988; Martin & Frayer, 2014; Wrangham, 2019). Once reputations from interaction histories are established, partners can reap steady gains from iterated cooperation (Andreoni & Miller, 1993; Kaplan et al., 2012, 2018; Kreps et al., 1982). However, established cooperators remain vulnerable to opportunistic exploitation by previously cooperative partners. These consequences have shaped our minds to detect and predict cooperators and cheaters in social contracts (Cosmides & Tooby, 1992; Green & Phillips, 2004). These adaptive problems continue to present themselves in modern society (Nowak & Sigmund, 2005; Seabright, 2010). Despite these challenges, cooperation is often achieved. We study cooperative behaviour prediction based on demographic beliefs, contextual clues and evidence of past behaviour.

We test the general hypothesis that people can rapidly forecast behavioural propensities under sparse information conditions such as upon first and second impressions of strangers. We also evaluate the general hypothesis that behaviour predictions improve as more information is made available for first and second impressions. Below we explain our experimental approach and detail our predictions that people inform their guesses about strangers by applying their prior demographic beliefs and available clues revealed by the target's description, appearance and behaviours.

We conducted a non-deceptive two-part study with financially motivated participants. In part one, across multiple rounds of play between matched partners, we recorded ‘thin-slice’ videos only a few seconds in duration (Ambady & Rosenthal, 1993) showing face-and-shoulder closeups of a university sample of participants taken just before their choices in each round of a ‘Split or Take All’ Prisoner's Dilemma (Prisoner's Dilemma) game variant with unknown end-game. In the second part of our study, we recruited online a set of raters to first make guesses about expected male and female cooperation rates from the Prisoner's Dilemma, then to guess the players’ Prisoner's Dilemma game behaviours. For each player guessed about, we provided a unique identification number and manipulated whether raters viewed either a thin-slice video showing the player, a photo still from the video, the player's self-identified gender label without photo or video or only the identification number. After forming a first impression, raters guessed each player's behaviour in the first round of gameplay. Raters also guessed behaviour in the second round after viewing first-round behavioural history and forming a second impression.

A unique feature of our thin slice and photo stimuli is that they feature contextually relevant information for the formation of first and second impressions. These stimuli may evoke relevant and difficult to fake signals that could diagnose behavioural propensity in the context of the player facing a social dilemma.

In social dilemmas like the repeated Prisoner's Dilemma, pursuing short-term non-cooperative benefits is at odds with the interests of developing cooperative partnerships. Despite the higher monetary rewards from successful non-cooperation, social dilemma experiments have demonstrated that cooperation can develop with unrelated strangers in one-shot environments (Balliet & Van Lange, 2013; Dawes & Thaler, 1988; Dickhaut et al., 2008; Kiyonari et al., 2000; McCabe et al., 1996, 2003; Ostrom & Walker, 2003; Schneider & Shields, 2022), finitely repeated games (Andreoni & Miller, 1993; Dawes & Thaler, 1988; Embrey et al., 2018; Mao et al., 2017) and infinitely repeated games with unknown endgame (Camera & Casari, 2009; Duffy and Ochs, 2009; van den Assem et al., 2012; Normann & Wallace, 2012). One explanation for this successful cooperation is that players can glean contextually evoked information and rely on accurate beliefs for predicting one another's game behaviour only moments later. The ability to predict cooperative behaviour from contextually relevant clues would also be valuable for navigating strategic interactions extending into the future, and therefore of great evolutionary significance since it could provide a basis for assortment.

Dawkins (1976) suggested that cooperation could evolve through self-assortment among conditional cooperators, if facilitated by a salient signal. He gave an example of a gene coding for a conspicuous ‘greenbeard’ phenotype with a propensity towards conditional cooperation; if those cooperators with greenbeard genes successfully self-assort, they can benefit from cooperation with one another and avoid exploitation by free riding, non-altruistic genes. However, as soon as non-altruists find a way to fake green beards, all bets are off for greenbeard fitness. Considering this problem, Price (2006) argued that greenbeard selection should be expected for reliable and relevant signals of cooperative propensity such as a behavioural history of cooperative behaviour. To this we add: when behavioural history is unavailable, reliable demographic information about a person revealed by their belonging to a population or gender, or perhaps revealed by their appearance, might also provide relevant signals of cooperative propensity.

When a population of players contains a mix of cooperative and uncooperative types, one might expect that players who have cooperative intentions will initially choose to cooperate and those with exploitative intentions will initially choose to cheat. For conditional cooperators who prefer cooperating when their partner is a cooperator, beliefs about the ratio of cooperators to cheater types in a population should be an important predictor of the strategies deployed in first-round interactions (Kiyonari et al., 2000). Upon first-impression, when no prior reputational information is available, one can apply their ‘homemade’ prior beliefs about the ratio of cooperators to non-cooperators likely to be encountered (Camerer & Weigelt, 1998) or derived from stereotyped assumptions about targets (Ames et al., 2012; McCabe et al., 2000). How those prior beliefs inform prediction strategies is less clear. One possibility is that forecasts are made using ‘probability matching’ strategies, where future outcomes are predicted with the frequency that approximately matches a prior belief or expected frequency. On average, probability matching tends to be less successful than using a pure optimisation strategy – predicting only the more expected outcome. While probability matching has been observed across various experiments, it tends to be less common under conditions like ours where participants are financially motivated and rewarded for correct predictions (Holt, 2007; Siegel et al., 1964; Siegel & Goldstein, 1959; Vulkan, 2000). From these considerations, we derive our first prediction: (P1) in the treatment where gender is not revealed, guesses of players’ Round 1 cooperativeness will be influenced by prior beliefs about cooperation propensity in the player population.

People expect behaviour in social dilemmas to vary by gender, and when players’ gender is revealed, people expect gender to be predictive of strategic behaviour (Fetchenhauer et al., 2010; Schniter & Shields, 2020; Sylwester et al., 2012). Across cultures, people expect that others’ tendencies to cooperate depend on their gender, with women characterised as generally more communal and cooperative than men (Eagly, 2009). Upon visual inspection, male and female gender is differentiated in less than a second (Fletcher-Watson et al., 2008), and usually achieving accuracy above 95% (Bruce et al., 1993; Bruce & Young, 2011; Hill et al., 1995; Jaeger et al., 2020). This suggests that descriptions and appearance revealing gender inform raters of gender-specific behavioural propensities that could be used for predicting Prisoner's Dilemma strategies that males and females deploy in interactions with strangers. Of course, to successfully apply beliefs about gender to predictions of strangers’ behaviour, their gender needs to be known and the beliefs about each gender need to be accurate. In treatments where raters know players’ gender, we expect that beliefs about gender influence guesses such that (P2) sufficiently correct gender beliefs are associated with more correct guesses.

When faces can be seen in photos (Fetchenhauer et al., 2010; Tognetti et al., 2013) or thin-slice video (Ambady et al., 2000; Ambady & Rosenthal, 1993; Fetchenhauer et al., 2010; Vogt et al., 2013), or during brief personal interaction (Brosig, 2002; DeSteno et al., 2012; Frank et al., 1993; Reed et al., 2012), first impressions are formed using the static or dynamic clues encountered (Snyder, 1984). Faces may communicate information about stable dispositional traits like cooperativeness (Fetchenhauer et al., 2010; Frank, 1988; Frank et al., 1993), and distinguishing characteristics like gender, formidability, health, kinship and ethnicity (Bruce et al., 1993; Fasolt et al., 2019; Zilioli et al., 2015). Facial displays of happiness and anger could also be helpful for behaviour prediction, as these displays are produced and understood by everyone, quickly interpreted – in well under a second (Batty & Taylor, 2003), and may be reliably informative of behavioural propensity (Ekman et al., 1987; Hirshleifer, 1987; Reed et al., 2012; Verplaetse et al., 2007). As facial clues can be diagnostic of cooperative propensity, and first impressions from appearances may sometimes be accurate (Fetchenhauer et al., 2010; Tognetti et al., 2013; Verplaetse et al., 2007; Vogt et al., 2013), we predict that (P3) guesses of Round 1 cooperativeness will be more accurate in treatments showing a photo or video of each player than in treatments not showing players’ appearances.

Brief in-person interactions and thin-slice videos of only a few seconds may reveal dynamic information about players that static photographs cannot (Ambadar et al., 2005; Harwood et al., 1999; Pike et al., 1997; Sato et al., 2004). This dynamic appearance information may help people make better predictions, but it could also present an unhelpful distraction. Dynamic faces may display ‘tells’, or involuntary facial cues, eye movements, blinking and brief micro-expressions, that can be used to assess the cooperative propensity of targets (Fetchenhauer et al., 2010; Frank, 1988; Frank et al., 1993; Hirshleifer, 1987; Reed et al., 2012). Dynamic faces may also reveal emotional expressivity, measured by the frequency and intensity of emotional expressions. Emotional expressivity can be used to index players’ likelihood of cooperation, as more emotionally expressive faces tend to be more cooperative (Schug et al., 2010). While expressive behaviour sampled in first impressions can improve judgmental accuracy (Ambady et al., 2000; Ambady & Rosenthal, 1993), it may not always be beneficial. Emotionally expressive faces are highly arousing and provocative stimuli, providing distraction that cannot be easily ignored (Palermo & Rhodes, 2007). If attention to faces is overly demanding of limited time or cognitive resources, the ability to make accurate behaviour predictions upon first impressions might be compromised. This possibility is consistent with distraction-conflict models of attention allocation (Baron, 1986; Durkin et al., 2020). Videos and in-person interactions that provide longer exposure to dynamic face stimuli may exacerbate this distraction problem. For example, Sylwester et al. (2012) asked raters to assess either thin-slice (1–5 s) or long (60–120 s) video clips of people playing a variation of the Prisoner's Dilemma (Prisoner's Dilemma) game, and to predict whether each player would choose ‘Split’ or ‘Take All’. Although they did not find that raters had above chance accuracy for long videos, they did find that accuracy was higher than expected by chance for the shorter thin slice videos. As the richer dynamic information from thin-slice videos may help form first and second impressions, we predict that (P4) guesses of Round 1 cooperativeness will be more accurate in the video treatment than in the photo treatment.

In repeated interactions, prior demonstrations of partners’ cooperative behaviour can help inform beliefs about their intentions to cooperate (Coricelli et al., 2000; McCabe & Smith, 2001). Even if first impressions are inaccurate, when new evidence of cooperative behaviour is revealed (e.g. after a round of game interaction), behaviour predictions based on informed second impressions may become more accurate (Andreoni & Petrie, 2008; Schniter & Shields, 2014, 2020).

**Figure 1.**
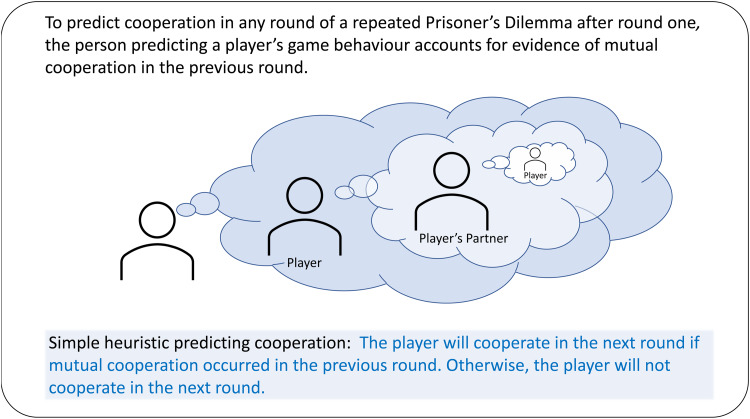
Conditional cooperation heuristic for predicting players’ cooperative propensity in a repeated Prisoner's Dilemma game with unknown endgame.

Players’ willingness to pursue cooperation conditionally depends on their preferences for mutual cooperation or exploitation, and consideration of whether partners previously cooperated (Kiyonari et al., 2000). This leads to selective cooperation among conditional cooperators, enabling conditional cooperators to escape exploitation and the consequential competitive disadvantage they would otherwise incur in repeated interactions with non-cooperators. After round one, we expect predictions of players’ behaviour to consider both players’ and partners’ previous behaviour. Figure 1 outlines a conditional cooperation heuristic that we expect people to apply when predicting cooperative behaviour. This simple heuristic expects conditional cooperators to rely on the tit-for-tat strategy (Rapoport et al., 1965) for selecting next round behaviours in the Prisoner's Dilemma, and for non-cooperators to consistently prefer non-cooperation. Tit-for-tat mutual cooperation does not explain the origins of the evolution of cooperation (Axelrod, 1984; Howard, 1988), but rather explains how, despite hazards from potential interactions with non-cooperators, conditional cooperation can be sustained given humans’ evolved capacity for reciprocal altruism among unrelated conspecifics (Trivers, 1971). To predict someone's likelihood to cooperate, people should be able to evaluate their history of cooperation and then apply this simple one-reason heuristic quickly, with little cognitive effort or demand for additional information. Selection is expected to have strongly favoured ‘fast and frugal’ heuristics such as the one we propose because of their efficiency, inferential speed and accuracy in decision-making situations constrained by limited information and available time (Gigerenzer & Goldstein, 1996; Hertwig & Herzog, 2009; Todd, 2001). In our experiment, round 2 guesses are made with knowledge of the players’ past round behaviours, while round 1 guesses are made with no past behaviours known. This leads us to predict that (P5) the guesses made about round 2 will be more accurate than guesses made about round 1.

Our paper proceeds as follows: in Section 2 we review background literature and compare our cooperative behaviour prediction study design to others. In Section 3 we provide methodological details, in Section 4 we present results, and in Section 5 we discuss the results, study limitations and extensions.

## 2. Background

A cheater and cooperator detection adaptation appears to have evolved for solving problems associated with social exchange and cooperation (Cosmides, 1989; Cosmides & Tooby, 1989, 1992, 2005). Accurate detection and prediction of cooperators and defectors is crucial for avoiding the pitfalls of interacting with non-cooperators or missing opportunities with cooperators (Cosmides & Tooby, 2005; Frank, 1988). Despite a small industry of research efforts to study cooperation prediction abilities, support for or against them has been unclear, in-part owing to a diversity of research designs.

A few studies find support for accurate game behaviour prediction (Brosig, 2002; Frank et al., 1993; Reed et al., 2012); however, others report mixed results with only partial support, or no support (Bonnefon et al., 2013, 2017; Efferson & Vogt, 2013; Fetchenhauer & Dunning, 2010; Jaeger et al., 2022; Kiyonari, 2010; Manson et al., 2013; Sparks et al., 2016; Sylwester et al., 2012; Tognetti et al., 2013; Verplaetse et al., 2007; Vogt et al., 2013). Several of these studies do not reward raters’ correct guesses (Sylwester et al., 2012; Tognetti et al., 2013; Verplaetse et al., 2007), which may negatively affect the accuracy of raters’ guesses. In our study, correct beliefs about each gender and guesses about individual players are incentivised with monetary rewards, which should motivate raters to make their best guesses (Smith, 1976). Cooperation prediction studies have also been limited to predictions of players with no reputational history of prior game behaviour. Our study is unique in that we study not only Round 1 guesses from first impressions with no reputational history, but also Round 2 guesses of those same players from informed second impressions – where raters know players’ behavioural history.

Many behaviour prediction studies draw raters and targets from the same subject pool. In some cases raters were shown targets that they had had prior interactions with or went on to play subsequent games with (Brosig, 2002; DeSteno et al., 2012; Frank et al., 1993; Manson et al., 2013; Reed et al., 2012; Sparks et al., 2016). Our worldwide online sample of raters is not drawn from the same local communities as the players they guess about, nor from the same convenience samples as the players, nor from among the set of players themselves. While convenient, more insular designs invite the possibility that prediction results are confounded by raters’ prior familiarity with targets, their involvement in the subject pool or experiment session, or behavioural norms specific to their local community.

Some have given attention to uncovering what aspects of targets’ appearance might be helping people make behaviour predictions (DeSteno et al., 2012; Jaeger et al., 2022; Manson et al., 2013; Reed et al., 2012; Tognetti et al., 2013), although none of these have examined how well people can otherwise predict gameplay in the absence of personal cues from photos, videos, and face-to-face interactions, for example, by asking the question, ‘in the absence of visual stimulus, could strangers’ gameplay be predicted with above-chance accuracy?’ Our study design allows us to answer this question. Of the game behaviour prediction studies that feature visual stimuli of players, many show images of the players under highly specific and unnatural conditions, such as where hair, clothes and colour are removed from faces or where faces are required to display emotionally neutral poses (Bonnefon et al., 2013; Jaeger et al., 2022). Other studies censor and manipulate the distributions of target characteristics to be equiprobable rather than varying naturally or representative of society's base rates (Oda et al., 2009; Olivola & Todorov, 2010). Yet other studies show videotapes of players, but drawn specifically from a disparate setting than where the game decision are predicted (Brown et al., 2003; Fetchenhauer et al., 2010). While many of these manipulations of visual images are ideal for increasing experimental control, for example to investigate the role of isolated player features (e.g. face shape or expressions) on rater predictions, they provide distinctly different approaches to studying behaviour prediction abilities that complicate a comparative interpretation of their results.

Our study does not feature photos and videos from contextually disparate or unnatural conditions, nor does it censor or manipulate distributions of target characteristics. While our design controls the experimental settings and methods of stimulus capture, we allow Prisoner's Dilemma participants to exhibit natural and ad libitum behaviour in the moments before the Prisoner's Dilemma game decision, when we capture their image.

## 3. Methods

Our study consists of two experimental procedures. In the first part, we use an experimental economic game and self-reported demographics to generate target stimuli consisting of thin-slice videos, facial photographs, identification numbers, gender labels and behavioural strategies from a participant sample of game players. In the second part of our study, we use an economic experiment to ask whether raters can predict players’ game behaviours based on beliefs about players, beliefs about male or female players, static and dynamic appearance, and behavioural history.

### 3.1. Stimuli from Prisoner's Dilemmas

First, we conducted a computerised laboratory procedure in an experimental economics laboratory using a ‘Split or Take All’ Prisoner's Dilemma game variant with an unknown end-game and anonymous unacquainted matched pairs. In the players’ instructions, we specified and explained a random-stopping rule to determine the chance of players continuing to another round: 4^1−*n*^ where *n* is the current round (e.g. the chance is 1/1, 1/4, 1/16, 1/64 for rounds 1–4, respectively). In those instructions, we clarify that players would interact for a minimum of two rounds, with the possibility of more rounds (see Appendix B for details).

Participants recruited to be ‘players’ in the Prisoner's Dilemma were randomly drawn from a subject pool of graduates and undergraduates at Chapman University. We used no deception and paid these players for the outcomes of their behaviour in the study. As such, all game decisions were incentivised by the economic consequences of the game. We ran 13 sessions, each taking approximately 60 min.

In this Prisoner's Dilemma each player chooses between ‘Split’ or ‘Take All’ strategies. Players were provided a payoff matrix explaining the consequences of both players’ choices (Table 1). If both players choose ‘Split’ they each get 5 dollars; if both choose ‘Take All’ they each get nothing. However, if one chooses ‘Split’ but not the other, the player choosing ‘Split’ gets nothing and the other player gets 10 dollars. In the classic Prisoner's Dilemma, non-cooperation strictly dominates cooperation, whereas here it weakly dominates cooperation: choosing ‘Take All’ can do at least as well, and sometimes better than choosing ‘Split’. One advantage of the Split or Take All variant is that the strategy labels used are intuitive because they directly describe the payoff goals.

**Table 1.** Split or Take All Prisoner's Dilemma game payoffs.

		Column	
		Split	Take All
Row	Split	5, 5	0, 10
	Take All	10, 0	0, 0

Note: Row, column player payoffs are in US dollars.

Ninety-four players aged 18–25 (51 men, 45 women) consented to be video recorded at intervals throughout the experimental procedure under standardised videographic conditions and for their recordings and experiment data to be made available for later research. Players were told that at no time would their or other players’ identities or video recordings be revealed to participants in their experiment session.

Videos of players were taken using computer display-mounted digital cameras in individual computer terminal cubicles, set at the same distance from uniform backgrounds. From the original video recordings capturing head-and-shoulder closeups with *ad libitum* behaviours and expressions in the 8 s directly preceding game decision making, we trimmed thin-slice videos 2–3 s in length without audio. Photographs showing the player were captured from the thin-slice video. For these photographs, we chose moments that best showed participants’ faces with screen-oriented gaze following conclusion of their statement.

### 3.2. Prisoner's Dilemma prediction experiment

We recruited 445 participants (Mean_age_=33.6, *S*tandard deviation_age_ = 12.0; 48.53% male, 48.98% female) using www.prolific.co. Participants were allowed from all countries and given up to 87 min to complete the experiment. We restricted recruitment to volunteers with normal or corrected-to-normal vision and English fluency and only allowed volunteers to participate in the study once. A total of 422 participants remained after excluding participants for violating requirements; specifically, we excluded (i) 11 for taking the survey on a smart phone despite prohibition against using small screen devices and (ii) 12 for completing the task in less than 480 s, a speed we considered to be humanly improbable. Table A1 reports the characteristics of these participants whom we refer to as raters.

All raters received instructions. To advance to the prediction study, raters had to complete, without error, a series of control questions verifying that a human responder is attentive to questions. Instructions and survey questions are available in the Online Appendix B.

Raters received the same instructions for the Prisoner's Dilemma that were provided to players in the first experimental procedure. Raters were informed that they would first make guesses about the Round 1 behaviours of the female and male players in the original study. For example, ‘On a scale ranging from 0% to 100% of the time, how often do you guess that females chose to ‘Split’ and ‘Take All’ in the first round of the original experiment’, with the requirement that these percentages must equal 100%. Raters answered identical questions about males. These guesses inform us of raters’ prior beliefs about female and male players. Next, raters made a series of guesses about the game behaviours of each player from the original study by selecting either the cooperative strategy (‘Split’) or the uncooperative strategy (‘Take All’) that they expected the player to have chosen. Each rater made these guesses about each of the 94 players. First, all guesses about Round 1 game behaviour were made. Next, with the history of each player and partner's Round 1 behaviour provided, raters made all guesses about Round 2 game behaviour. We used no deception and paid raters for the accuracy of their guesses in the study. As such, all guesses made were incentivised by the economic consequences of their accuracy using a quadratic scoring rule if the guess is within 1/6 of the actual value.

### 3.4. Treatment groups

**Figure 2.**
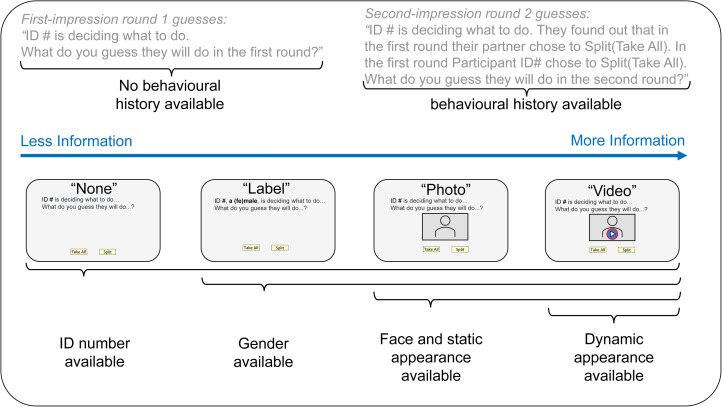
Schematic diagram showing incremental manipulation of gender, static and dynamic appearance, and behavioural history information available for first and second impressions in a cooperative behaviour prediction experiment.

We conducted a 4 × 1 between-subjects design with raters randomly assigned to the treatment cells. There are four treatments manipulating player information available for first and second impressions. We call these ‘None’ (*n* = 108), ‘Label’ (*n* = 101), ‘Photo’ (*n* = 108) and ‘Video’ (*n* = 105). Our study began April 2021 with ‘None’, ‘Photo’ and ‘Video’ treatments and added the ‘Label’ treatment August 2022. All treatments make player IDs available and manipulate availability of behavioural history within-subjects. No history is available for raters’ first-impression Round 1 guesses and the history of players’ and partners’ Round 1 choices is available for raters’ second-impression Round 2 guesses. The Label, Photo and Video treatments reveal gender. The Photo and Video treatments reveal static player appearance. Only the Video treatment reveals dynamic appearance. As such, this design incrementally manipulates the availability of information about gender, static appearance, dynamic appearance and contextualised behavioural history (see Figure 2), allowing us to systematically evaluate the general hypothesis that availability of more of this information for first and second impressions leads to better predictions. This design also allows us to test predictions about the role of raters’ prior beliefs (P1), beliefs about genders (P2), players’ appearance (P3), static vs. dynamic appearance (P4) and behavioural history (P5). We preregistered our treatments at aspredicted.org (#61202, #103594) before collecting their data. Internal review board approval was granted by Chapman University (#1718H016, #1314H065).

### 3.4. Measurements and analyses

Statistical analysis was performed using Stata/SE 17.0. We measure beliefs about players’ cooperation propensity as continuous variables in the 0–100% range. We evaluate the accuracy of beliefs using ‘belief error’: the absolute difference between the belief and the actual player behaviour. We measure and evaluate raters’ predictions of players’ individual choices that we call ‘guesses’, as well as performance across all predictions in a round. For each individual choice we measure the rater's binary guess, either ‘Split’ or ‘Take All’, and if the guess is correct: 1 if yes, 0 if no.

To measure accuracy over many predictions, we use signal detection theory, which evaluates the raters’ ability to distinguish potential cooperation from defection (Green & Swets, 1966; Macmillan & Creelman, 2004). Signal detection theory critically distinguishes two theoretically independent constructs: accuracy and bias. In our prediction task, accuracy is the raters’ ability to discriminate cheaters who choose ‘Take All’ from cooperators who choose ‘Split’, while bias is the raters’ tendency to guess players who choose ‘Take All’ or ‘Split’, independent of their ability to discriminate cheaters from cooperators. These signal detection theory constructs are based on the cooperator detection rate (*H*) and the cheater detection rate (*R*). *H* measures the proportion of times the rater guesses correctly given that the players choose ‘Split’ and *R* measures the proportion of observations the rater guesses correctly given the players choose ‘Take All’. Using these rates, ‘accuracy’ is operationalised as [*Z*(*H*) −  *Z*(1 − *R*)], where a zero value indicates that the rater shows no demonstrable ability to distinguish cooperators from cheaters. That is, a zero value indicates guess correctness is neither better nor worse than chance. ‘Bias’ is operationalised as −0.5 [*Z*(*H*) +  *Z*(1 − *R*)], where negative values represent a bias towards guessing ‘Split’, and positive values represent a bias towards guessing ‘Take All’. The function *Z*(.) is the inverse of the standard normal cumulative distribution, which converts rates into *Z-*scores. We transform rates of zero to 1/100,000 and rates of one to 99,999/100,000, so that the *Z-*scores do not go to infinity. Two alternative measures of accuracy, ‘correctness’ and the ‘odds ratio’ are described in Appendix C.

For first-round guesses about unknown gender players in the mixed gender population, we calculate raters’ ‘belief about players’ cooperative propensity’ from an average of their beliefs about male and female players.

To assess whether raters with more accurate beliefs make more correct guesses, we create dummy variables for belief accuracy that code for what we call ‘sufficiently correct beliefs’. The dummy is 1 if the rater's belief about a gender is greater than or equal to 50% and players of that gender tended to be cooperative, or if the rater's belief about a gender is less than 50% and players of that gender tended to be non-cooperative. Otherwise, the dummy is 0. A sufficiently correct dummy helps us evaluate whether correct beliefs could contribute to more correct guesses. If raters tend to base their guesses on sufficiently correct beliefs, then average guess correctness should increase with sufficiently correct beliefs. We also consider an alternative measure of gender-based belief accuracy, the ‘absolute error of belief’, measured as the absolute value of the difference between the belief and average player cooperation in round 1.

To evaluate differences in measures over summary statistics, we use *Dependent* 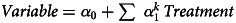 as the regression model and report the Wald test statistic for where the treatment dummies are equal. All significantly reported results are robust using the nonparametric Kruskal–Wallis test.

When evaluating the effects of treatment groups and controls on raters’ individual guesses, we use logit panel regression, which controls for dependencies of repeated observations of the same rater. Panels identify the raters and trials identify the players.
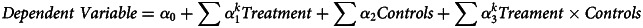


When the dependent variable is bounded within the unit interval, as with beliefs, or when we can reject that the dependent variable is normally distributed using the Shapiro–Wilk test, as with accuracy, we use a generalised least squares regression.

When evaluating the accuracy change between the first and second rounds, we use the general least squares panel regression, which controls for dependencies of repeated observations of the same rater. Panels identify the raters, and trials identify the rounds. Accuracy measures the raters’ ability to discriminate cheaters from cooperators and is constructed using all 94 guesses made in the round.
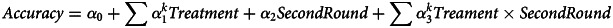


## 4. Results

Among Prisoner's Dilemma players we can observe the endogenous emergence and natural distribution of cooperative behaviours among matched pairs and the effects of game interaction outcomes on subsequent game behaviour. Below we describe the results of our Prisoner's Dilemma prediction study, which elicited raters’ beliefs about male and female players’ cooperativeness in the Prisoner's Dilemma, followed by predictions about individual Prisoner's Dilemma players’ game behaviour based on first and second impressions. On average, raters completed the study procedure in 24.4 min and earned $4.56. Prediction response times per target by treatment are reported with rater demographics in Table A1. Signal detection measures including cooperator detection rate, cheater detection rate, accuracy and bias are reported by treatment in Table A2.

### 4.1. Stereotypes about Prisoner's Dilemma players’ cooperation rates

**Figure 3.**
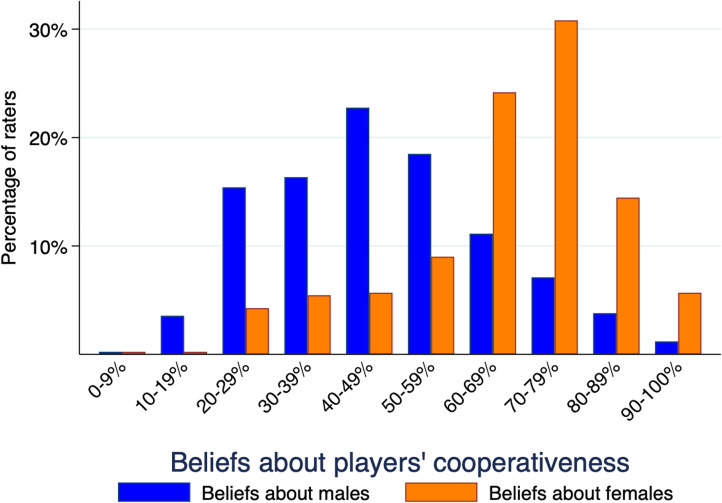
Raters’ gender-specific beliefs about the proportion of cooperative male and female players in the first round of a repeated Prisoner's Dilemma game with unknown endgame.

Raters’ beliefs about players indicate that they expected players to cooperate 54% of the time in the first round (Table 2). There were no significant differences in the belief about players between treatments (χ^2^(3) = 3.99, *p* = 0.262). Male and female raters’ beliefs about players did not differ significantly (χ^2^(1) = 0.10, *p* = 0.746). Male players were believed to be less cooperative (44.2%) than females (63.9%). Since we find no significant difference in beliefs over treatments, we combine treatments and find that gender-specific beliefs about male and female players were heterogeneous (Figure 3), significantly correlated (Pearson 0.503, *p* < 0.001), and significantly different (Wilcoxon matched-pairs signed-rank test, *Z* = 16.1, *p* < 0.001).

**Table 2.** Raters’ prior beliefs about players’ cooperativeness.

	Belief about male players	Belief about female players	Belief about all players
Belief revealed by:			
Male rater	46.3	61.8	54.1
*N* = 206	(18.6)	(18.4)	(16.6)
Female rater	42.2	66.5	54.4
*N* = 206	(17.7)	(15.2)	(13.8)
All other raters	40.3	50.4	45.4
*N* = 10	(13.6)	(18.7)	(15.3)
Combined	44.2	63.9	54.0
*N* = 422	(18.1)	(17.2)	(15.3)
Actual cooperativeness:	Male players	Female players	All players
Round 1	61.4	86.0	74.5
	(49.2)	(35.1)	(43.8)
Round 2	43.2	62.0	53.2
	(50.1)	(49.0)	(50.2)
Both rounds	52.3	74.0	63.8
	(50.2)	(44.1)	(48.2)

Note. Where beliefs are reported, values are mean percent of time (standard deviation in parentheses) that raters guess that each gender chooses ‘Split’ in Round 1 of the repeated Prisoner's Dilemma. Where players actual cooperativeness is reported, values are mean percent of time (standard deviation in parentheses) players choose ‘Split’.

To evaluate beliefs, we use the regression *Belief error* =  *α*_0_ +  *α*_1_*Rater gender* +  *α*_2_*Belief Gender* +  *α*_3_*Rater Gender*  × *Belief Gender*, where belief error is the difference between a gender-specific belief and the gender-specific Round 1 observed behaviour, each rater is the panel, and the two beliefs are the trials. These gender beliefs significantly underestimated actual male player cooperation (61.4%) and female player cooperation (86.0%) in the first round (males: χ^2^(1) = 153.16, p < 0.001; females: χ^2^(1) = 392.56, p < 0.001). Male raters believed males to be slightly more cooperative (46.3%) than female raters (42.2%), a significant difference (χ^2^(1) = 5.46, p < 0.019). Similarly, female raters believed females to be more cooperative (66.5%) than male raters (61.8%), a significant difference (χ^2^(1) = 7.44, *p* < 0.006).

### 4.2. First-impression guesses about Prisoner's Dilemma players’ Round 1 game behaviour

Upon exposure to stimulus describing and sometimes showing Prisoner's Dilemma players deciding how to play in Round 1 of a repeated Prisoner's Dilemma, raters made rapid first impressions and predictions of each of 94 players, averaging across treatments 1.2 (None), 1.4 (Label), 3.1 (Photo) and 8.3 (Video) seconds per player. Consistent with their beliefs, raters underestimated Round 1 cooperation in all treatments, predicting 58.7% cooperation, when it was 74.5% (Table 3, panel A, all χ^2^(1) > 159.29, all *p* < 0.001).

**Table 3.** Raters’ guesses about players’ cooperative behaviour.

*Panel A: Summary statistics*			
Treatment (N)	Guessed split	Average correct	Accuracy
*Round 1 guesses (actual Split = 74.5%)*			
None	63.6	56.1	−0.115
(108)	(27.2)	(14.0)	(0.546)
Label	61.0	58.7	0.238
(101)	(15.9)	(8.6)	(0.363)
Photo	56.1	55.9	0.214
(108)	(16.8)	(8.7)	(0.348)
Video	54.0	53.4	0.113
(105)	(13.2)	(8.5)	(0.362)
All	58.7	56.0	0.110
(422)	(19.4)	(10.4)	(0.436)
Difference	16.77***	13.49**	48.40***
*Round 2 guesses (actual Split = 53.2%)*			
None	60.0	60.2	0.536
(108)	(18.4)	(8.2)	(0.451)
Label	60.8	60.7	0.561
(101)	(14.6)	(7.9)	(0.438)
Photo	59.0	62.5	0.674
(108)	(12.1)	(6.3)	(0.351)
Video	55.4	61.6	0.606
(105)	(11.1)	(6.5)	(0.352)
All	58.8	61.3	0.595
(422)	(14.5)	(7.3)	(0.402)
Difference	8.58*	6.55	7.42

*Panel B: Raters’ second-round guesses and average correct by players’ behavioural history.*						
		Raters’ Round 2 guess Split				
Behavioural history	Actual Split	None	Label	Photo	Video	Combined
Both take all	83.3	40.0	38.3	31.9	33.8	36.0
Take All/partner Split	22.2	38.6	34.7	24.0	22.2	29.8
Split/partner Take All	26.3	25.9	32.2	31.4	27.9	29.3
Both split	70.6	82.6	83.4	84.8	79.9	82.7
Total	53.2	60.0	60.8	59.0	55.4	58.8
		Raters’ Round 2 average correct				
	Percentage of players	None	Label	Photo	Video	Combined
Both take all	6.4	43.4	41.1	39.4	41.0	41.2
Take All/partner Split	19.1	55.7	59.8	66.0	65.6	61.8
Split/partner Take All	20.2	61.0	58.8	60.2	61.2	60.3
Both Split	54.3	63.4	64.1	64.9	62.8	63.8
Total	100	60.2	60.7	62.5	61.6	61.3

Note: Values for guessed Split, correctness, and actual Split are percentages. Standard deviations are in parentheses. Difference reports the results of the Wald test that the treatment dummy coefficients in generalised linear model regression are equal: chi-squared with 3 degrees of freedom reported; *** *p* < 0.001, ** *p* < 0.01, * *p* < 0.05. These test results are robust using Kruskal–Wallis.

Below, we evaluate our research questions concerning the predicted effect of beliefs, labels, photos, and videos on Round 1 game behaviour guesses.

#### Where gender cannot be detected, are Round 1 guesses influenced by beliefs about cooperation propensity in the player population (P1)? Yes

In the None treatment, where the raters did not know the players’ gender, players are expected to cooperate 55.9% of the time according to raters’ *beliefs.* Raters *guessed* that 63.6% would cooperate in Round 1. The effect of *belief* on guesses is significant in the None treatment (χ^2^(1) = 189.75, *p* < 0.001) (Table 4, regression 1).

**Table 4.** First-round guesses and correctness controlling for the raters’ beliefs. 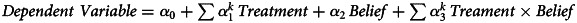

Dependent variable	(1) Guess Split for all treatments	(2) Guess Split when gender revealed	(3) Correct guess when gender revealed
Label	2.28***		
	(4.91)		
Photo	1.77***	0.52**	0.09
	(3.99)	(3.46)	(1.34)
Video	2.41***	1.30***	0.06
	(5.32)	(8.78)	(0.84)
Belief about players	0.08***		
	(13.77)		
Label × belief about players	−0.04***		
	(−5.47)		
Photo × belief about players	−0.04***		
	(−4.66)		
Video × belief about players	−0.05***		
	(−6.46)		
Gender-specific belief		0.05***	
		(30.47)	
Photo × gender-specific belief		−0.01***	
		(−5.04)	
Video × gender-specific belief		−0.03***	
		(−12.89)	
Sufficiently correct stereotype			0.83***
			(14.88)
Photo × sufficiently correct stereotype			−0.28***
			(−3.78)
Video × sufficiently correct stereotype			−0.39***
			(−5.22)
Constant	−3.69***	−2.37***	−0.19***
	(−11.09)	(−21.24)	(−3.78)
Guess	39,668	29,516	29,516
Raters	422	314	314
Log-likelihood	−24,026	−18,057	−19,882
Akaike information criteria	48,070	36,128	39,779
Bayesian information criteria	48,147	36,186	39,837
Chi-squared (7/5/5 degrees of freedom)	338***	1,861***	438***

*Z-*Value in parentheses. *** *p* < 0.001, ** *p* < 0.01. We report the results of logit regression, where the rater is the panel and players are the trials. Regression (1) includes first-round data from all treatments. Regressions (2) and (3) include first-round data only from treatments where players’ gender is revealed: Label, Photo and Video. The variable Belief about players in regression (1) refers to the average of the rater's male and female gender-specific beliefs. Gender-specific beliefs in regression (2) refers to the applicable belief about male or female player given the player's self-described gender. ‘Sufficiently correct stereotype’ in regression (3) is a dummy variable that equals one if the rater's gender stereotype is ≥50% and players of that gender tended to be cooperative, or if the rater's gender stereotype is <50% and players of that gender tended to be non-cooperative.

#### Are Round 1 guesses influenced by gender-specific beliefs such that sufficiently correct beliefs predict correct guesses in treatments where players’ gender is labelled or seen? (P2) Yes

The effect of gender-specific beliefs on guesses is positively significant in the Label, Photo, and Video treatments where gender can be visually detected (all χ^2^(1) > 258.98, all *p* < 0.001) (Table 4, regression 2). The effect of gender-specific beliefs is significantly stronger for the Label treatment than the Photo treatment (χ^2^(1) = 25.42, *p* < 0.001), and the effect is significantly stronger for the Photo treatment than for the Video treatment (χ^2^(1) = 65.85, *p* < 0.001).

Guess correctness is influenced by belief accuracy (Table 4, regressions 3). For all treatments, sufficiently correct beliefs are significantly positively correlated with correct guesses (all χ^2^(1) > 74.78, all *p* < 0.001). These results remain robust when using the ‘absolute error of belief’ measure: errors are significantly negatively correlated with correct guesses in all treatments (allχ^2^(1) > 34.37, all *p* < 0.001).

#### Are Round 1 guesses more accurate in the treatments showing the player's appearance (P3)? Yes

We report accuracy by treatment controlling for the round in Table 5. Prediction accuracy is improved for treatments showing players’ appearance (χ^2^(1) = 6.39, *p* = 0.015). We test for robustness using alternative operationalisations of accuracy described in Appendix C. This result is robust to tests using correctness but not the odds-ratio (Table C1).

**Table 5.** Accuracy by treatment controlling for the round. 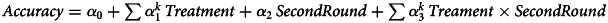

Label	0.35***
	(6.30)
Photo	0.33***
	(5.96)
Video	0.23***
	(4.11)
Second round	0.65***
	(11.80)
Label × second round	−0.33***
	(−4.13)
Photo × second round	−0.19*
	(−2.44)
Video × second round	−0.16*
	(−2.01)
Constant	−0.11**
	(−2.95)
*N*	844
Raters	422
Log-likelihood	−434.94
Akaike information criterion	886
Bayesian information criterion	924
Chi-squared (7 degrees of freedom)	359.40***

*Z-*Value in parentheses. *** *p* < 0.001, ** *p* < 0.01, * *p* < 0.05. All general least squares regressions use measures constructed over the round where the rater is the panel and rounds are the trials. All results are robust using ordinary least squares regression.

#### Are Round 1 guesses more accurate in the video treatment than in the photo treatment (P4)? No

Round 1 accuracy is not statistically more accurate in the Video treatment than in the Photo treatment (χ^2^(1) = 3.14, *p* = 0.076). This result is consistent with tests using correctness and the odds-ratio (Table C1).

### 4.3. Second-impression guesses of Prisoner's Dilemma players’ Round 2 game behaviour

Raters guessed 58.8% of players would cooperate in Round 2, quite close to their guess of 58.7% cooperation in Round 1 (Table 3, panel A). Players’ cooperative behaviour decreased from 74.5% in Round 1 to 53.2% in Round 2. Compared with Round 1 guesses, accuracy increased for Round 2 guesses (Table 3, panel A). Below we report results that help explain these performance improvements.

#### Are round 2 guesses more accurate than round 1 guesses (P5)? Yes

**Figure 4.**
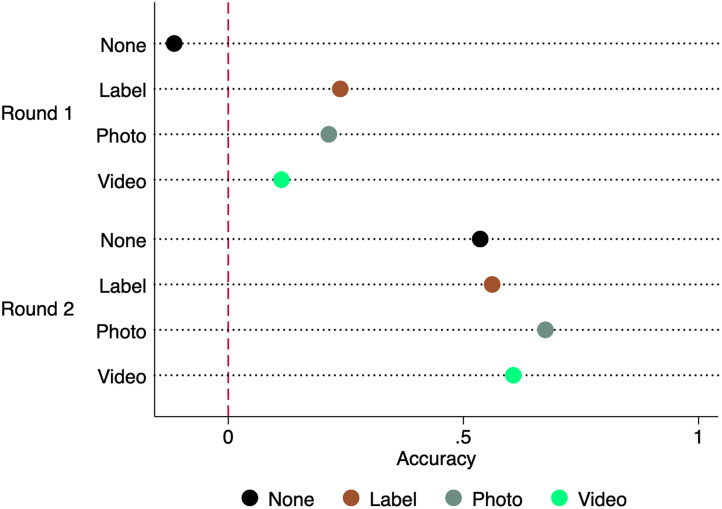
Accuracy of first-round and second-round guesses by treatment. Accuracy is measured as [*Z*(*H*) −  *Z*(1 − *R*)], where *Z*(.) is the *Z-*score, *H* is the cooperator detection rate and *R* is the cheater detection rate. An accuracy value of zero is no better or worse than chance and indicates no demonstrable ability to distinguish cooperators from cheaters.

Across treatments, Round 2 guesses were significantly more accurate than in Round 1 for all treatments (all χ^2^(1) > 32.09, all *p* < 0.001). This result is robust to tests using correctness and odds-ratio (Table C1). Figure 4 illustrates that Round 2 guesses are more correct than expected by chance (accuracy > 0) across treatments (all *p* < 0.001). Next, we conduct post-hoc analysis to determine whether the artefactual conditions endogenously created by players’ Round 1 behavioural history affected raters’ Round 2 guess performance, and whether players’ facial description and appearance may have played a role.

### 4.4. Post-hoc analyses of Round 2 guesses given beliefs, conditions with gender or appearance revealed, and behavioural history

Round 2 guess accuracy improves significantly across conditions (all χ^2^(1) > 32.09, all *p* < 0.001). However, Round 2 guess accuracy was significantly greater in conditions revealing players’ appearance (Photo and Video), than in conditions that did not (χ^2^(1) = 5.42, *p* = 0.019).

Raters’ Round 2 guesses vary across the players’ four possible behavioural histories: ‘Both Take All’, ‘Take All/Partner Split’, ‘Split/Partner Take All’ and ‘Both Split’ (Table 3, panel B). Compared with the Round 1 guesses, raters significantly increased their Round 2 guesses of cooperation for the ‘Both Split’ behavioural history condition, and significantly decreased their guesses of cooperation for behavioural history conditions where at least one partnered player chose ‘Take All’.

Round 2 guesses are affected by seeing gender labels or players’ appearance in the context of Round 1 behavioural history, as these clues help improve guess correctness about male players generally (Table A2), and guess correctness for all players in the behavioural conditions where one or both partners chose ‘Take All’ (Table A3). We find significant differences in correctness when raters had access to players’ appearances, but no significant differences between the Photo and Video treatments. When raters see a player's appearance in a ‘Take All/Partner Split’ interaction, they more aptly guess player behaviour in Round 2. Likewise, when raters see the player's appearance in a ‘Split/Partner Take All’ interaction, they more aptly guess Round 2 behaviour – resulting in more correctness than in conditions without the player's appearance (Table A3).

## 5. Discussion

These results provide supporting evidence for the mechanisms designed to rapidly predict others’ cooperativeness when forming first and second impressions. Below we discuss the importance of prior demographic beliefs, contextual clues and evidence of past behaviour for revealing behaviour prediction abilities.

Our results suggest that when the incentive structure of a game is easily understood, raters can make cooperation predictions easily and in rapid succession, taking about 3–4 s on average to form impressions, evaluate, and guess about each player. More often than not, these predictions are correct – even though the players are strangers and the raters initially have no direct behavioural evidence of past behaviours. The incentive structure we chose for our ‘Split or Take All’ Prisoner's Dilemma game appears to be widely understood – leading to common perspectives and expectations among players and raters; it is identical to that of games featured on television shows such as *Friend or Foe*, *Golden Balls* or *Take It All* which since have been analysed as a natural experiment of cooperation (Burton-Chellew & West, 2012; van den Assem et al., 2012). In *Friend or Foe*, *Golden Balls* or *Take It All* games, players choose ‘Split’ 53% of the time, and young adult males are less cooperative than young adult females (Burton-Chellew & West, 2012; van den Assem et al., 2012). Our raters expected males to be less cooperative, and for players to cooperate 54% of the time, almost identical to the game show average.

While we investigate the possibility that there are some reliably observable signals among players with propensity to cooperate, for example, visual clues of player gender that correspond to raters’ gender-specific beliefs, our results do not suggest cooperators are detectable ex-ante owing to visual greenbeard-like signals from facial expression or expressivity that might distinguish individuals as being Round 1 cooperators or non-cooperators. As our results suggest, accurate cooperation detection in Round 1 relies on guesses about a large set of players based on fairly accurate prior beliefs. As such, upon first impressions, first-round cooperators are detectable at a rate better than expected by chance, but with error. This agrees with others’ findings that participants correctly expect that most other people in experiments with them are cooperative (Andreoni & Miller, 1993; McCabe et al., 2000), consistent with observed cooperation rate evidence (Andreoni & Miller, 1993; Camerer & Weigelt, 1998; Hayashi et al., 1999; Kiyonari et al., 2000; Kurzban & Houser, 2005; McKelvey & Palfrey, 1992). A closer look at the decomposed Round 1 cooperator detection rate and Round 1 cheater detection rate indicates that raters correctly identify 59.8% of cooperators but only 44.8% of non-cooperators, with a bias value of −0.316 indicating a tendency towards predicting cooperation (Table A2). The better-than-chance correct guesses are consistent with raters’ applying their beliefs that most people are cooperators. Raters predicted that around 55 of the 94 players (~58.7%) would choose to cooperate in Round 1 and, indeed, most (70 of 94 or 74.5%) players chose to cooperate in Round 1. As our results demonstrate, the positive correlation between prior beliefs about Round 1 cooperation rates in the general population (54%), or for males (44.2%) and females (63.9%) specifically, and the observed Round 1 cooperation rates (61.4% males, 86.0% females, 74.5% all players) explains much of why this better-than-chance prediction of cooperative behaviour exists.

Interestingly, the effect of correct gender-specific beliefs on correct guesses of a player's Round 1 behaviour is strongest in the gender label treatment. For Round 1 guesses, there is no evidence that observable signs from players’ static or dynamic appearances improve cooperation detection beyond their contribution to informing raters of the player's gender. Our gender-label treatment allows us to carefully isolate the effect of male or female gender from other effects of visual appearance available to raters in photo and video treatments. While all our Prisoner's Dilemma players self-identified as either male or female, a small portion of our raters chose to not identify as male or female. Future studies will benefit from inquiry into the alternative gender identities and concepts that are becoming increasingly preferred by survey respondents and might better reveal gender influences if carefully measured (Snyder et al., 2022).

The informational differences afforded by our treatments suggest that raters may not have equal reason to rely on gender-specific beliefs across treatments. Across conditions that reveal gender, the Label treatment provides raters less player information than the Photo treatment, which provides less player information than the Video treatment. As a result of these differences in available information, raters may trade off the value of gender clues for additional visual clues. An additional concern about differences across these treatments is that the appearance of static or dynamic faces may present an unhelpful distraction for raters who might be better off relying on accurate prior beliefs. The formation of first impressions from faces may be so automatic and non-conscious that they are relied upon even when objectively better information is available (Olivola & Todorov, 2010; Rezlescu et al., 2012) or when it is known that one should avoid being influenced by faces (Blair et al., 2004; Hassin & Trope, 2000; Palermo & Rhodes, 2007). While raters in our study appear to be trading off the influence of gender-specific beliefs for additional appearance information, the effect of more appearance information on Round 1 guess accuracy is unhelpful: the Photo treatment is somewhat less accurate than the Label treatment, and the Video treatment is no better off, consistent with the conflict-distraction model. As we discuss further below, the effect of appearance information on second impressions is positive, improving all measures of accuracy relative to those treatments with no player appearance revealed.

Across first- and second-round predictions, accuracy in the Video treatment is no better than in the Photo treatment. Attention to dynamic faces requires more time and attentional resources potentially distracting or interfering with processing capacity for tasks separate from face inspection (Lavie, 1995; Pessoa et al., 2002). The attentional costs and longer response times in our Video treatment may have contributed to a greater conflict-distraction effect, producing less guess correctness and accuracy than in the Photo treatment. Our research design did not compel standard response times across treatments to control for these costs. More research is needed to understand the reasons for response time variation and the role of response time costs.

Upon learning the details of players’ Round 1 Prisoner's Dilemma interactions, raters can form second impressions with the behavioural history information gleaned. From these second impressions, raters make better-than-chance predictions of players’ Round 2 Prisoner's Dilemma game behaviours across all treatments – improving their guess performance from Round 1 guesses. Our proposed behaviour prediction heuristic (Figure 1) may help explain how people use behavioural history information to ‘mind read’ the propensities of others, effectively predicting their cooperative behaviours in mixed-motive social dilemmas (Baron-Cohen, 1997; McCabe & Smith, 2001; Sylwester et al., 2012). Future research will be able to demonstrate the role of behavioural history on guess accuracy by experimentally manipulating behavioural history information availability for Round 2 guesses. For second impressions, player appearance also helps raters make more accurate guesses: guess accuracy is higher in photo and video treatments – a result which was not seen with Round 1 guesses. Round 2 guesses are also more correct under conditions where one partner chose ‘Take All’. Prior research suggests that more masculine male faces are associated with perceptions of aggressiveness and dominance (Geniole et al., 2015; Sell et al., 2009; Zilioli et al., 2015), consistent with the idea that males who appear stronger and more masculine have greater potential bargaining power via coercive formidability and therefore can be expected to act more aggressively, reactively, and less cooperatively in social dilemma interactions (Daly & Wilson, 1988; Sell et al., 2012). Given our effects of male faces on Round 2 guesses, it may be productive for future research to investigate further how variation in male cues, such as facial masculinity and formidability, may be predictive of cooperativeness in repeated games, especially in the context of game interactions with previously non-cooperative partners, where entitlement and reactive anger may be at play.

Raters demonstrate better-than-chance behaviour prediction abilities in three out of four treatments for Round 1 guesses, and in all the treatments for Round 2 guesses. Our results suggest that these prediction abilities respond to sparse clues, like gender and appearance, available in first and second impressions. As gender identity and photo or video appearance are influential parameters in self-presentation across a variety of human interaction media affecting investment, voting, legal decisions, hiring, mate selection and cooperative interaction (Snyder et al., 2022; Todorov, 2017), our results provide important insight into key hazards and trade-offs involved with revealing or not revealing gender identity and static or dynamic appearance when first or second impressions form and new relationships develop.

Our study provides an explanation for why cooperation is so commonly observed among strangers in social dilemmas like the Prisoner's Dilemma despite incentives to be uncooperative: people can predict the cooperation propensities of most other people and likely use this ability to identify and maintain mutually beneficial cooperative relationships. Prior studies demonstrated that cheater detection abilities are particularly sensitive to rule violation information (Brown & Moore, 2000; Cosmides, 1989; Cosmides & Tooby, 1992; Fiddick & Erlich, 2010; Oda et al., 2006). Our study demonstrates that cheater and cooperator detection is sensitive to sparse person and context information, adding another facet to our understanding of how cheater detection adaptations are designed. Our study also provides insight into accurate predictions of trust re-extension, an important but precarious and all-too-common problem in personal and business relationships (Robinson & Rousseau, 1994; Schniter & Sheremeta, 2014).

The behavioural sciences have extensively studied the design of people's chosen behaviours in potentially cooperative strategic interactions. However, clean experimental tests and a clear understanding of people's expectations of others’ behaviours in unacquainted and repeated interactions have been missing. The evidence presented here suggests people can accurately predict the cooperativeness of strangers, helping explain the broad extent of human cooperativeness revealed by experimental and ethnographic studies. In conclusion, our study provides further support for the claim that an evolutionary–functional framework is a productive and promising approach to uncovering the nature of human cooperation and cooperative behaviour prediction.

## Supporting information

Schniter and Shields supplementary material Schniter and Shields supplementary material

## Supplementary material

The supplementary material for this article can be found at https://doi.org/10.1017/ehs.2023.30.

## Research transparency and reproducibility

Experiment stimuli along with complete details of the Prisoner's Dilemma game, procedure and stimulus development are openly available online at https://doi.org/10.5281/zenodo.4321821. The data that support the findings of this study are openly available at https://doi.org/10.5281/zenodo.7465288.
