# Supplementary material for: Better-than-chance prediction of cooperative behaviour from first and second impressions
Source: Evol Hum Sci. 2024 Jan 8;6:e2. doi: 10.1017/ehs.2023.30 (PMC10955359; doi:10.1017/ehs.2023.30)
Supplement: Schniter and Shields supplementary material [file S2513843X23000300sup001.docx]

**Online Supplementary Materials for “Better-than-chance Prediction of Cooperative Behaviour.”**

**Appendix A**

Table A1. Rater demographics.

| **[A]** | Guess response time in seconds per target: | |  | Self-identify with: | | |  | | Prior familiarity with: | | | |
| --- | --- | --- | --- | --- | --- | --- | --- | --- | --- | --- | --- | --- |
| Treatment (N) | First Impression – Round 1 | Second impression – Round 2 | Age in years | Male | Female | Other or prefer not to answer | |  | | Players in image | University students | University |
| None (108) | 1.16 (1.05) | 2.21 (1.49) | 36.3 (12.7) | 47.2 | 50.0 | 2.8 | |  | | N/A | 0.9  (9.6) | 2.8  (16.5) |
| Label (101) | 1.40  (0.64) | 2.40 (1.44) | 32.8 (11.3) | 50.5 | 45.5 | 4.0 | |  | | N/A | 1.0  (9.9) | 3.0  (17.1) |
| Photo (108) | 3.13 (2.56) | 3.70 (2.39) | 33.6 (11.8) | 49.1 | 50.0 | 0.9 | |  | | 0.9  (9.6) | 0.9  (9.6) | 0.9  (9.6) |
| Video (105) | 8.32 (2.39) | 8.82 (3.21) | 32.6 (12.3) | 48.6 | 49.5 | 1.9 | |  | | 0.0  (0.0) | 0.0 (0.0) | 1.9  (13.7) |
| Total (422) | 3.50 (3.43) | 4.28 (3.49) | 33.9 (12.1) | 48.8 | 48.8 | 3.6 | |  | |  | 0.7  (8.4) | 2.1  (14.4) |

| **[B]** | Raters’ country of origin | | | | | | | | | | | | | | | | | | | | | | | | | | | | | | | | | | |  |
| --- | --- | --- | --- | --- | --- | --- | --- | --- | --- | --- | --- | --- | --- | --- | --- | --- | --- | --- | --- | --- | --- | --- | --- | --- | --- | --- | --- | --- | --- | --- | --- | --- | --- | --- | --- | --- |
| Treatment | Austria | Bangladesh | Cambodia | Canada | China | Germany | Hong Kong | Hungary | India | Indonesia | Ireland | Israel | Italy | Jordan | Kenya | Libya | Luxembourg | Mexico | Namibia | Nepal | Netherlands | Pakistan | Philippines | Poland | Russian Federation | Saudi Arabia | Serbia | South Africa | Switzerland | Thailand | United Kingdom | United States | Vietnam | Data unavailable | Total | |
| None |  |  |  | 20 | 2 | 1 |  |  |  |  |  |  | 1 |  |  |  |  |  | 1 |  |  | 1 | 1 |  | 1 |  |  | 1 |  |  | 57 | 22 |  |  | 108 | |
| Label |  |  | 1 | 21 | 1 |  |  |  |  |  | 1 |  |  |  |  |  |  |  |  |  | 1 |  | 3 | 1 |  |  |  |  | 1 | 1 | 52 | 17 |  | 1 | 101 | |
| Photo |  |  |  | 22 | 3 |  |  | 1 | 1 |  |  |  | 1 |  |  |  | 1 |  |  | 1 |  |  |  |  |  |  | 1 | 1 |  |  | 48 | 24 |  | 4 | 108 | |
| Video | 1 | 1 |  | 34 | 2 |  | 2 |  |  | 1 | 1 | 1 |  | 1 | 1 | 1 |  | 1 |  | 1 |  |  |  |  | 1 | 1 |  |  |  |  | 37 | 15 | 1 | 2 | 105 | |
| Total | 1 | 1 | 1 | 97 | 8 | 1 | 2 | 1 | 1 | 1 | 2 | 1 | 2 | 1 | 1 | 1 | 1 | 1 | 1 | 2 | 1 | 1 | 4 | 1 | 2 | 1 | 1 | 2 | 1 | 1 | 194 | 78 | 1 | 7 | 422 | |

Note: Panel A reports raters’ average response time in seconds per target, raters’ self-identified gender as mean percentages, and mean percentages of raters with responses indicating prior familiarity with experimental stimuli sources (see online Appendix B for survey question details). Standard deviations are in parentheses. Panel B reports frequency of birthplace that raters’ report as their country of origin.

Table A2. Signal detection measures of raters’ cooperative behaviour prediction for first and second rounds of a repeated prisoner’s dilemma game with unknown endgame.

| Treatment (N) | Cooperator Detection (*H*) | Cheater Detection (*R*) | Accuracy | Bias |
| --- | --- | --- | --- | --- |
| *Round 1 Guesses* | | | | |
| None | 63.2 | 56.1 | -0.115 | -0.480 |
| (108) | (27.4) | (14.0) | (0.546) | (1.131) |
| Label | 63.2 | 58.7 | 0.238 | -0.277 |
| (101) | (15.8) | (8.6) | (0.363) | (0.548) |
| Photo | 58.1 | 55.9 | 0.214 | -0.149 |
| (108) | (16.5) | (8.7) | (0.348) | (0.619) |
| Video | 55.0 | 53.4 | 0.113 | -0.087 |
| (105) | (13.9) | (8.5) | (0.362) | (0.381) |
| All | 59.8 | 56.0 | 0.110 | -0.316 |
| (422) | (19.5) | (10.4) | (0.436) | (1.169) |
| *Round 2 Guesses* | | | | |
| None | 68.9 | 50.2 | 0.536 | -0.268 |
| (108) | (20.4) | (19.7) | (0.451) | (1.089) |
| Label | 70.3 | 49.9 | 0.561 | -0.374 |
| (101) | (15.5) | (17.9) | (0.438) | (0.777) |
| Photo | 70.2 | 53.8 | 0.674 | -0.227 |
| (108) | (12.9) | (14.5) | (0.351) | (0.361) |
| Video | 66.0 | 56.6 | 0.606 | -0.131 |
| (105) | (12.9) | (12.9) | (0.352) | (0.312) |
| All | 68.9 | 52.6 | 0.595 | -0.249 |
| (422) | (15.8) | (16.6) | (0.402) | (0.714) |
| Note: Values for cooperator detection, and cheater detection are percentages. Standard deviations are in parentheses. | | | | |

Table A3: Round 2 guesses and correctness controlling for behavioural history and treatments with appearance.

| $Dependent variable= \alpha_{0}+ \alpha_{1} Appearance+ \sum\alpha_{2}^{h} History+ \sum\alpha_{3}^{h} History\times Appearance$ | | | | |
| --- | --- | --- | --- | --- |
| Dependent variable | Guess |  | Correct |  |
| Both Take All | -2.45 | *** | -0.83 | *** |
|  | (-24.55) |  | (-9.79) |  |
| Take All/Partner Split | -2.52 | *** | -0.33 | *** |
|  | (-36.66) |  | (-6.07) |  |
| Split/Partner Take All | -3.23 | *** | -0.10 |  |
|  | (-44.02) |  | (-1.93) |  |
| Faces | -0.15 |  | 0.02 |  |
|  | (-1.23) |  | (0.52) |  |
| Both Take All X Appearance | -0.03 |  | -0.14 |  |
|  | (-0.26) |  | (-1.44) |  |
| Take All/Partner Split X Appearance | -0.38 | *** | 0.33 | *** |
|  | (-4.73) |  | (5.17) |  |
| Split/Partner Take All X Appearance | 0.54 | *** | -0.06 |  |
|  | (6.58) |  | (-0.99) |  |
| Constant | 1.91 | *** | 0.56 | *** |
|  | (18.37) |  | (15.63) |  |
| N | 39,668 |  | 39,668 |  |
| Groups | 422 |  | 422 |  |
| Log-Likelihood | -19,176.8 |  | -26,140.2 |  |
| Akaike information criterion | 38,371.6 |  | 52,298.3 |  |
| Bayesian information criterion | 38,448.8 |  | 52,375.6 |  |
| Chi-Square (7 degrees of freedom) | 9,817.0 | *** | 514.3 | *** |

Z-value in parentheses. ***: p < .001. Logit regression results reported; the rater is the panel and players are the trials. The condition ‘Both Split’ is the baseline. Appearance is equal to one if the treatment is Photo or Video.

**Appendix B**

# Experimental Task Instructions and Survey Questions

A unique set of questions is being generated that will test your eligibility to complete this study. Participants who answer these questions incorrectly are NOT eligible, will be screened out immediately, and will NOT receive any payment.

What did you see?

*[One of the images below was randomly selected and displayed]*

- A blue rectangle and black oval
- A green cross and orange triangle
- A red circle and green star
- A yellow triangle and red circle
- A black oval and blue cross
- A pink square and purple star

What is the answer to this question?

*[One of four randomly selected word problems, with unique answer below, would appear here]*

- Six
- Five
- Twelve
- Ten

## Consent

We invite you to participate in a research study being conducted by Eric Schniter and Timothy Shields, both professors from Chapman University. The purpose of the study is to understand individuals’ ability to predict. If you agree to participate, we would like you to complete tasks that involve making guesses and answering survey questions. The study will take on average 30 minutes to complete. Your payment will be your wage and a bonus. The bonus will be as high as $3 depending on the accuracy of your predictions. At the end of the study, you will learn your task performance and the associated payment amount. You will receive payment for completing the study in its entirety. There is minimal foreseeable risk associated with this study. All responses are anonymous. Taking part in this research study is completely voluntary. If you do NOT wish to participate in this study, you can exit the study anytime. However, incomplete responses cannot be used for research and therefore, you will NOT receive payment. If you have any questions about the study, please contact Eric Schniter. If you have any questions about your rights as a research participant, please contact the Human Subjects Office at (714) 628-2833. Thank you very much for your consideration of this research study. Select the appropriate option below to indicate whether you agree to participate.

- Yes, I agree to participate in this study
- No, I do NOT agree to participate in this study

## Introduction

This survey is part of an experiment in the economics of decision-making. Various research agencies have provided funds for this research. By following the instructions carefully and making good decisions, you may earn an additional amount of money besides the payment for completing the survey. The actual amount of additional money that you may earn will depend on your decisions. Your earnings will be reported to you after you complete the survey.

There are a couple of basic rules you must follow:

1. Do not communicate with others, or allow others to assist you while completing the survey.
2. Do not discuss this survey with others after completing the survey.
3. If you have any questions, comments, or concerns please communicate them to the researchers running this study.

### General Description of the Survey

The survey is broken into four separate parts. All four parts must be completed to earn payment. After you finish you will be provided with a Completion ID. In the first, second, and third parts of the survey, one randomly chosen guess determines your additional earnings from that part. Your overall income from the survey will be based on the sum of earnings from the first three parts of the survey and from your wage for completing all sections of the survey. It is in your best interest to make a careful decision in all possible situations. Researchers at Chapman University have previously conducted an experiment using an anonymous economic interaction (between a randomly paired Person 1 and Person 2) over a computer network. Participants in the original experiment earned money based on the interactions of their choices. Today’s survey will ask you to make guesses about what participants did in that original experiment. A description of that experiment follows.

**IMPORTANT**: You will NOT participate in the experiment explained below, but it is important that you understand it because you will make guesses related to people and decisions from this original experiment conducted at Chapman University. Below are the instructions that were provided to participants in that original experiment.

### The First-Round Decision *[hover over text]*

In this experiment you will be randomly paired with one other person. The other person, like yourself, was recruited from Chapman University. During this experiment you will be paired with this person through a computer network. At no time will your true identity be revealed to the other participants here today. Even though we are video recording for research purposes, during this experiment your video recorded image will never be transmitted or shown to other participants in today’s experiment.

The choices made by both you and the other person will affect how a $10 dollar jackpot will be allocated. Because these choices affect your earnings, you should make a deliberate and conscious choice. You can either choose *Split* or you can choose to *Take All*. The other person you are paired with makes the same choice. If BOTH you and the person you are paired with choose *Split* you will both get a payoff of $5. If you BOTH choose to *Take All* you will both get a payoff of $0. If you choose to *Take All* but the other person chooses *Split* you will get a payoff of $10 and the other person will receive $0. Likewise, if you choose *Split* but the other person chooses to *Take All,* then you receive $0 and the other person receives $10. These payoffs are summarized in the table below. The bold number in each quadrant of the box below is the payment received by you, the other non-bold number is the payment received by the other person:

When making your choice, you will not know the choice made by the other person. We first will ask you to state your intention while being recorded. That is, we want you to make a non-binding statement about what you intend to do, *Split* or *Take All*. Next, we ask you to make your choice: *Split* or *Take All*. After everyone in the experiment has made their choice, the computer will report the results: your choice, the choice made by the other person, and your payoffs. After reviewing the results, you will be asked to complete a short survey before moving on.

#### Set of Multiple Rounds

You will participate in a set composed of more than a single round. Each round is the same. The number of rounds that you will participate in is determined probabilistically by the computer. After each round has been finished, the probability of participating in another round is (1/4)^(n-1) where n is the number of rounds so far. Since (1/4)^0 = 1 there will be at least 2 rounds and some probability of future rounds. However, it is uncertain how many more rounds there will be beyond these first 2 rounds. In all rounds you will interact with the same person that you were paired with in the first round.

#### Reminder

#### You will be participating in a set of two or more rounds and interacting with the same other person for all rounds. Even though we are video recording for research purposes, you are guaranteed that during this experiment your video recorded image will never be transmitted or shown to other participants in today’s experiment.

## SITUATION UNDERLYING TODAY’S SURVEY

Display This Question:

If Treatment = None or Label

Researchers have collected extensive data from the original experiment that was just described and are now interested to learn more about what you think participants did in the experiment.  After being presented with a participant identification number and any relevant information about previous round choices and outcomes, you will have the opportunity to guess whether the participant went on to choose “*Split*” or “*Take All*” during that round. A correct guess can earn you $1.00 and an incorrect guess can earn you $0.00. You will have the opportunity to make 188 guesses across 3 parts of the survey. With a guess randomly chosen for payment from each part, you can earn a maximum of $3.00 in addition to the wage you are guaranteed to earn by completing this survey.

Display This Question:

If Treatment = Photo

Researchers have collected extensive data from the original experiment that was just described and are now interested to learn more about what you think participants did in the experiment. We have prepared images (photos from videos) of the participants during each of their multiple rounds of interaction, taken moments after they stated their intentions, but before making their choice to “*Split*” or “*Take All*.” We will present each of these photos to you (one at a time). Reminder: participants never saw any images or videos of each other. After being presented with a participant identification number, a photo of a participant from the original experiment, and any relevant information about previous round choices and outcomes, you will have the opportunity to guess whether the participant went on to choose “*Split*” or *Take All*” during that round. A correct guess can earn you $1.00 and an incorrect guess can earn you $0.00. You will have the opportunity to make 188 guesses across 3 parts of the survey. With a guess randomly chosen for payment from each part, you can earn a maximum of $3.00 in addition to the wage that you are guaranteed to earn by completing this survey.

Display This Question:

If Treatment = Video

Researchers have collected extensive data from the original experiment that was just described and are now interested to learn more about what you think participants did in the experiment. We have prepared short videos of the participants during each of their multiple rounds of interaction, taken moments after they stated their intentions, but before making their choice to “*Split*” or “*Take All*.” We will present each of these videos to you (one at a time). Reminder: participants never saw any images or videos of each other. After being presented with a participant identification number, a video of a participant from the original experiment, and any relevant information about previous round choices and outcomes, you will have the opportunity to guess whether the participant went on to choose “*Split*” or “*Take All*” during that round. A correct guess can earn you $1.00 and an incorrect guess can earn you $0.00. You will have the opportunity to make 188 guesses across 3 parts of the survey. With a guess randomly chosen for payment from each part, you can earn a maximum of $3.00 in addition to the wage that you are guaranteed to earn by completing this survey.

You will learn the total reward from correct guesses after completing all guesses and responding to a final set of questions. Starting on the next page, you will always have the option to review the description of the original experiment by hovering your mouse over the phrase below

**Or**i**ginal Experiment Description**

*[When the mouse hovered over the bold text above, the text from The First-Round Decision block would appear as an overlay]*

## Survey Part 1: gender guesses

**Original Experiment Description**

*[When the mouse hovered over the bold text above, the text from The First-Round Decision block would appear as an overlay]*

Males and females (self-identified) participated anonymously in the original experiment. In the first rounds of interaction in the original experiment, before they discovered what their partners did, how often did females choose “split” or “take all” and how often did males choose “split’ or “take all”? Your guesses will be compared to the observations from the original experiment.

Your guess can earn you as much as $1.00 so long as it is within 16.7%, above or below, the observed frequency of the target(s)’ behavior.

On a scale ranging from 0% to 100% of the time, how often do you guess that **females** chose to “split” or to “take all" in the first round of the original experiment? Complete the following statements according to your expectations by choosing values that total 100%.

_______ % of the time females chose “take all.”

_______ % of the time females chose “split.”

On a scale ranging from 0% to 100% of the time, how often do you guess that **males** chose to “split” or to “take all" in the first round of the original experiment? Complete the following statements according to your expectations by choosing values that total 100%.

_______ % of the time males chose “take all.”

_______ % of the time males chose “split.”

Congratulations, you have finished Part 1. Next, you will make guesses about what participants did in their *first-round* interactions with matched partners. A correct guess can earn you $1.00. Ready to go to Part 2?

## Survey Part 2: *first round* guesses

*[This choice was looped over the 94 target players randomly]*

# of 94

**Original Experiment Description**

*[When the mouse hovered over the bold text above, the text from The First-Round Decision block would appear as an overlay]*

Display This Question:

If Treatment != Label

**ID#** is deciding what to do. What do you guess they will do in the first round?

Display This Question:

If Treatment = Label

**ID#** a **<gender>** is deciding what to do. What do you guess they will do in the first round?

Display This Question:

If Treatment = Photo

<insert photo>

Display This Question:

If Treatment = Video

<insert video – options below would not appear until the video ended>

| - Take All | - Split |
| --- | --- |

Congratulations, you have finished Part 2.

Next, you will be able to see what participants did in the *first-round* interactions and make guesses about what they do in their *second-round* interactions. A correct guess can earn you $1.00. Ready to go to Part 3?

## Survey Part 3: *second round* guesses

*[This choice was looped over the 94 target players randomly]*

# of 94

**Original Experiment Description**

*[When the mouse hovered over the bold text above, the text from The First-Round Decision block would appear as an overlay]*

Display This Question:

If Treatment != Label

**ID#** is deciding what to do is deciding what to do.

They found out that in the *first* round their partner chose to **<split/take all>.**

In the first round Participant **ID#** chose to **<split/take all>.**

What do you guess they will do in the second round?

Display This Question:

If Treatment = Gender

**ID #X,** a **<gender>** is deciding what to do is deciding what to do.

They found out that in the *first* round their partner chose to **<split/take all>.**

In the first round Participant **ID#** chose to **<split/take all>.**

What do you guess they will do in the second round?

Display This Question:

If Treatment = Photo

<insert photo>

Display This Question:

If Treatment = Video

<insert video – options below would not appear until the video ended>

| - Take All | - Split |
| --- | --- |

Congratulations, you have finished Part 3.

In the next part of the survey, we ask you to answer a final set of questions, required for your completion of the survey. Ready to go to Part 4?

## Survey Part 4

What is your age? [input number]

Which gender do you identify with? [male, female, other, don’t want to answer]

Before providing you feedback on your guess, we present to you a final set of statements concerning men and women and their relationships in contemporary society. Please indicate the degree to which you agree or disagree with each statement. Your answers will not affect your rating or payment.

- No matter how accomplished he is, a man is not truly complete as a person unless he has the love of a woman.
- Many women are actually seeking special favors, such as hiring policies that favor them over men, under the guise of asking for "equality."
- In a disaster, women ought not necessarily to be rescued before men.
- Most women interpret innocent remarks or acts as being sexist.
- Women are too easily offended.
- People are often truly happy in life without being romantically involved with a member of the other sex.
- Feminists are not seeking for women to have more power than men.
- Many women have a quality of purity that few men possess.
- Women should be cherished and protected by men.
- Most women fail to appreciate fully all that men do for them.
- Women seek to gain power by getting control over men.
- Every man ought to have a woman whom he adores.
- Men are complete without women.
- Women exaggerate problems they have at work.
- Once a woman gets a man to commit to her, she usually tries to put him on a tight leash.
- When women lose to men in a fair competition, they typically complain about being discriminated against.
- A good woman should be set on a pedestal by her man.
- There are actually very few women who get a kick out of teasing men by seeming sexually available and then refusing male advances.
- Women, compared to men, tend to have a superior moral sensibility.
- Men should be willing to sacrifice their own well-being in order to provide financially for the women in their lives.
- Women, as compared to men, tend to have a more refined sense of culture and good taste.

*[All questions answered with 6-point Likert: disagree strongly, disagree somewhat, disagree slightly, agree slightly, agree somewhat, agree strongly]*

Before participating in today's study, were you familiar with Chapman University?

- No. I had no familiarity with Chapman University.
- Yes, I was familiar with Chapman University before, but I don't have personal connections to it.
- Yes, I was familiar with Chapman University and have personal connections to it.

Before participating in today's study, were you familiar with Chapman University’s students?

- No. I had no familiarity with Chapman University's students.
- Yes, I was familiar with Chapman University's students before, but I don't have personal connections to them.
- Yes, I was familiar with Chapman University’s students and have personal connections to them.

Display This Question:

If Treatment = Photo

Or Treatment = Video

Have you ever seen any of the people shown in today's study before today?

- No. I did not recognize any of the people shown in the survey as people I have seen before today.
- Yes, I recognized a person or people shown in the survey as someone I have seen before today.

# Payment Feedback

Based on the sum of your earnings from a randomly chosen guess in Part 1, a randomly chosen guess in Part 2, and a randomly chosen guess in Part 3 of the survey, you earned $X in addition to your fixed payment for survey completion.

**Appendix C**

**Alternative operationalisations of accuracy.**

At the individual level, we measure the rater’s binary guess (i.e., “Split” versus “Take All”) and if the guess is correct (i.e., 1 if yes, 0 if no). To measure accuracy over many raters’ predictions, we use the signal detection theory constructs of “accuracy” and bias throughout our paper (see section 3.4 for details). This accuracy measure evaluates the raters’ ability to discriminate cheaters who choose “Take All” from cooperators who choose “Split”, and bias measures raters’ tendency to guess players choose “Take All” or “Split”, independent of their ability to discriminate cheaters from cooperators. (Green & Swets, 1966; Macmillan & Creelman, 2004). Alternative accuracy measures exist, including ‘correctness’ and the ‘odds-ratio’. We describe how each of these is operationalized below, then present a table comparing these alternative measures by treatment controlling for the round, with our featured ‘accuracy’ measure from signal detection theory.

Correctness is the average correct guess over rounds. While the measures is simple to calculate, correctness does not measure how much better than chance prediction is. For example, assume players chose “Split” 75% of the time. Both rater A and rater B can have correctness scores of 75% despite having used different prediction strategies. Rater A guessed “Split” 80% of the time: identifying 86.66% of players who indeed chose “Split” and 60% of players who did not. Rater B guessed “Split” 100% of the time, demonstrating no ability to distinguish cheaters choosing “Take All” from cooperators choosing “Split”, despite having the same correctness score as rater A by chance.

The odds-ratio measures how much better predictions are than expected by chance alone. Formally this is defined by taking the number of correct guesses in a round divided by the expected number of correct guesses that would be due to chance alone, $\alpha\beta+(1-\alpha)(1 -\beta)$, where $\alpha$ is the proportion of time that the rater guesses “Split” and $\beta$ is the proportion of the time that players choose “Split”. Better-than-chance correctness is defined by odds-ratio values greater than one. Using the example above, rater A’s odds-ratio is $75\%\div65\%\cong1.154$, while rater B’s is $75\%\div75\%=1$.

Returning to the example and consideration of our “accuracy” and bias measures from signal detection theory, rater A has a higher accuracy score of 0.857 and bias of -.682, showing some ability to distinguish cheaters from cooperators and a lower tendency to over-predict players choose “Split” compared to rater B, who has the lowest possible accuracy score of zero and the maximum bias score of -4.265 toward over-predicting players choose “Split.” With recognition of the important role that player Split/Take All ratios have on different raters’ guess correctness, we prefer the diagnostic features of the accuracy and bias measures informed by signal detection theory to evaluate raters guesses, how much better those guesses are at correctly predicting player behavior than expected by chance, and how rater bias contributes to guess accuracy.

**Table C1: Alternative measures of accuracy by treatment controlling for the round with** **tests for robustness of results across measures.**

$$DV=\alpha_{0}+\sum\alpha_{1}^{k}Treatment+\alpha_{2}SecondRound+\sum\alpha_{3}^{k}Treament\times SecondRound$$

| DV | Correctness |  | Odds-Ratio |  |
| --- | --- | --- | --- | --- |
| Label | 0.03 | * | 0.07 | *** |
|  | (2.06) |  | (4.32) |  |
| Photo | -0.00 |  | 0.07 | *** |
|  | (-0.19) |  | (4.20) |  |
| Video | -0.03 | * | 0.04 | * |
|  | (-2.24) |  | (2.30) |  |
| SecondRound | 0.04 | *** | 0.20 | *** |
|  | (3.35) |  | (12.13) |  |
| Label X SecondRound | -0.02 |  | -0.06 | ** |
|  | (-1.12) |  | (-2.59) |  |
| Photo X SecondRound | 0.03 |  | -0.02 |  |
|  | (1.53) |  | (-0.84) |  |
| Video X SecondRound | 0.04 | * | -0.00 |  |
|  | (2.41) |  | (-0.10) |  |
| Constant | 0.56 | *** | 0.99 | *** |
|  | (66.00) |  | (85.89) |  |
| Log-Likelihood | -849.91 |  | -593.33 |  |
| Chi-Squared (7 degrees of freedom) | 97.27 | *** | 499.87 | *** |
| P3Result: None + Label < Photo + Video | 10.06 | ** | 2.21 |  |
| P4Result: Video $≯$ Photo | 4.21 |  | 3.50 |  |
| P5Result: Round2 > Round1for each treatment | all > 4.27 | * | all > 65.96 | *** |

Z-value in parenthesis. ***: p < .001, **: p < .01, *: p < .05. All general least squares regressions use measures constructed over the round where the rater is the panel (422) and rounds are the trials (2 per rater). All tests for robustness of results are based on Chi-squared tests with 1 degree of freedom.
